# Prenatal maternal psychological distress and the risk of autism spectrum disorders in offspring: results from a meta-analysis of observational studies

**DOI:** 10.3389/fpsyg.2026.1682620

**Published:** 2026-04-20

**Authors:** Dan Lin, Zhiqing Chen, Yanxia Wang, Qinfang Qian, Ping Ou, Jingmin Guo

**Affiliations:** Fujian Maternity and Child Health Hospital, College of Clinical Medicine for Obstetrics & Gynecology and Pediatrics, Fujian Medical University, Fuzhou, China

**Keywords:** ASD, autism, autism spectrum disorders, maternal, offspring, pregnancy, prenatal, psychological distress

## Abstract

**Introduction:**

Psychological distress, such as stress, depression or anxiety, is a prevalent mental health concern during pregnancy. However, data on the association between prenatal maternal psychological distress and the risk of autism or autism spectrum disorders (ASD) in their offspring have not been synthesized systematically. We performed a meta-analysis to explore this issue and provide evidence regarding maternal mental health screening and ASD prevention.

**Methods:**

Six electronic databases were systematically searched up to June 2025. English-language full-text observational studies were included, with no geographic or race restrictions. Studies that quantitatively assessed the association between maternal psychological distress during pregnancy and the risk of ASD in offspring were eligible for inclusion. Pooled odds ratios (ORs) were calculated. Heterogeneity, publication bias, and sensitivity analyses were assessed.

**Results:**

Among 484 full-text records screened, 22 studies were eligible. Data analysis demonstrated that offspring of mothers with prenatal psychological distress have a 72% higher likelihood of being diagnosed with ASD or autism after the age of two (OR = 1.72, 95% CI 1.50–1.97, *p* < 0.01) compared to those of mothers without distress. This association was observed across different study designs and ASD diagnostic ascertainment methods, although effect estimates varied. Substantial between-study heterogeneity was observed (*I*^2^ = 87.90%), largely attributable to differences in study design, ASD ascertainment and distress assessment rather than psychological distress subtype.

**Conclusion:**

In this meta-analysis, prenatal maternal psychological distress was associated with an increased likelihood of an ASD diagnosis in offspring. Across the included studies, effect estimates were generally similar for stress, depression, and anxiety, despite substantial heterogeneity in study design and exposure assessment. This consistency suggests that elevated ASD risk is not confined to a single diagnostic category of maternal distress. At the same time, the findings should be interpreted considering the variability in how psychological distress was measured and controlled for across studies. Taken together, the results indicate that maternal psychological distress during pregnancy warrants attention in epidemiological research and routine antenatal care, without implying that specific psychiatric subtypes can be clearly distinguished in terms of offspring ASD risk.

**Systematic review registration:**

https://www.crd.york.ac.uk/PROSPERO/view/CRD420251119825, PROSPERO: CRD420251119825.

## 1 Introduction

Autism spectrum disorder (ASD) is a neurodevelopmental condition characterized by differences in social interaction and communication, as well as repetitive behaviors. Epidemiological data indicated that ASD prevalence has risen dramatically from 0.2% in the late 20th century to 2.8% in 2022 (Fombonne, 2003; Maenner et al., 2020; Salari et al., 2022). This growth rate significantly surpassed what can be attributed solely to diagnostic criteria changes and improved clinical recognition, suggesting environmental factors may contribute substantially to ASD pathogenesis and triggering a systematic exploration of environmental risk factors. Among these, maternal psychological distress during pregnancy has emerged as a key focus in ASD etiology research due to its high prevalence and potential for preventive intervention. Recent syntheses of ASD etiology have increasingly emphasized a developmental-origins framework, in which adverse conditions during gestation and the perinatal period, such as abnormal gestational length, low birth weight, neonatal jaundice, and obstetric complications, form a coherent network of risk factors acting through placental, endocrine, and inflammatory pathways. Within this framework, maternal physiological and psychological states during pregnancy are viewed as upstream regulators of fetal neurodevelopmental programming (Jenabi et al., 2023a; Jenabi et al., 2023b; Jenabi et al., 2024; Salehi et al., 2024).

Existing literature identified prenatal psychological distress in pregnant women as comprising stress, depression, anxiety, etc. (Wu et al., 2020). Data from the World Health Organization’s Global Burden of Disease Study indicated that 18.7–25.3% of pregnant women exhibit clinically significant psychological symptoms, with this prevalence demonstrating an upward trend concurrent with accelerating urbanization and mounting social pressures (Gavin et al., 2005; Biaggi et al., 2016; Woody et al., 2017; World Health Organization, 2022). Prenatal psychological distress, being a prevalent mental health concern during pregnancy, has accumulated substantial biological evidence regarding its effects on fetal neural development. Experimental evidence demonstrates that maternal stress disrupts fetal nervous system development through multiple biological pathways, including hypothalamic–pituitary–adrenal (HPA) axis activation, placental epigenetic modifications, and maternal systemic inflammatory responses (McGowan and Matthews, 2018; Kassotaki et al., 2021; Champagne et al., 2024; Dieckmann and Czamara, 2024). Molecular analyses further indicate that dysregulated glucocorticoid receptor expression and elevated pro-inflammatory cytokine levels may impair neurogenesis and synaptogenesis (Webster et al., 2001; Xavier et al., 2016; Sevilla et al., 2021). Different forms of psychological distress during pregnancy, including stress, depression, and anxiety, are clinically heterogeneous and may differ in duration and severity. However, studies examining biological correlations of prenatal distress have repeatedly reported involvement of similar neuroendocrine, inflammatory, and epigenetic processes. Whether these partly shared mechanisms nonetheless give rise to meaningful differences in offspring neurodevelopmental outcomes across distress subtypes remains insufficiently resolved.

Notably, several of the biological mechanisms proposed for prenatal psychological distress overlap with those implicated in other established perinatal risk factors for ASD. For instance, dysregulation of the placental–fetal HPA axis has been discussed as a potential pathway linking abnormal gestational timing, including post-term birth, to later ASD risk (Jenabi et al., 2023a; Jenabi et al., 2023b). Endocrine, immune, and inflammatory alterations have likewise been reported in association with neonatal risk profiles such as low birth weight and other perinatal complications (Salehi et al., 2024). In this context, maternal psychological distress may be considered as one of several prenatal conditions capable of influencing similar biological systems during gestation, rather than as a distinct or isolated exposure within the perinatal risk landscape of ASD.

Prenatal psychological distress has frequently been examined in relation to ASD risk, but results from observational studies have not been consistent. While some studies report elevated risks, others have found weak or null associations. Differences in study design, the way ASD was defined, and how maternal distress was assessed are often noted when comparing these findings, but their relative contribution to the observed variability has not been formally evaluated. Consequently, it remains difficult to determine whether the reported heterogeneity reflects true etiological differences or methodological variation across studies. To address this issue, we undertook a meta-analysis to estimate the overall association and to explore whether differences in distress subtype and assessment approach were associated with variation in effect estimates.

## 2 Methods

### 2.1 Search strategy

We searched for observational studies that investigated the association between prenatal maternal psychological distress exposure and the risk of autism or ASD in their offspring and had quantitative results. According to the previous study, psychological distress includes stress, anxiety, and depression that are not classified as clinical mental disorder (Wu et al., 2020). A systematic literature search was developed in electronic databases (PubMed, EMBASE, Web of Science, PsychINFO, Scopus, and Cochrane Library) in accordance with PRISMA guidelines, with no starting date restriction and extending through June 30, 2025, to identify studies reporting quantitative associations between maternal psychological distress during pregnancy and offspring’s autism or ASD risk (Moher et al., 2010). The studies presented the outcomes of effect sizes as odds ratios (ORs), risk ratios (RRs) or hazard ratios (HRs). Initial development of the search strategy was performed in PubMed, with subsequent adaptations applied to all other databases. Identification of relevant literature was conducted through utilization of the following conceptual themes and keywords: (1) psychological distress: stress, anxiety or depression; (2) autism: autism, autism spectrum disorder or ASD; (3) maternal exposure period: pregnancy, gestation, perinatal, prenatal, peripartum or antenatal; (4) time for measuring health outcome: in children/adolescence/young people, during childhood, in offspring. The four themes were integrated using the Boolean operator “AND.” Subsequently, manual screening of references from eligible studies was performed to identify additional relevant literature.

### 2.2 Eligibility criteria

Observational studies that met the following criteria were included in this meta-analysis: (1) quantitatively examined the effect size of the association between maternal psychological distress exposure and the risk of autism or ASD in their offspring; (2) limited the psychological distress that only occurred during pregnancy; (3) applied a clear definition for the measurement(s) of autism or ASD and the age of diagnosis (e.g., DSM-V, ICD-10; 6.8 ± 3.6 years, 2–6 years); (4) inclusion was restricted to peer-reviewed, full-text articles published in English.

**Figure 1:**
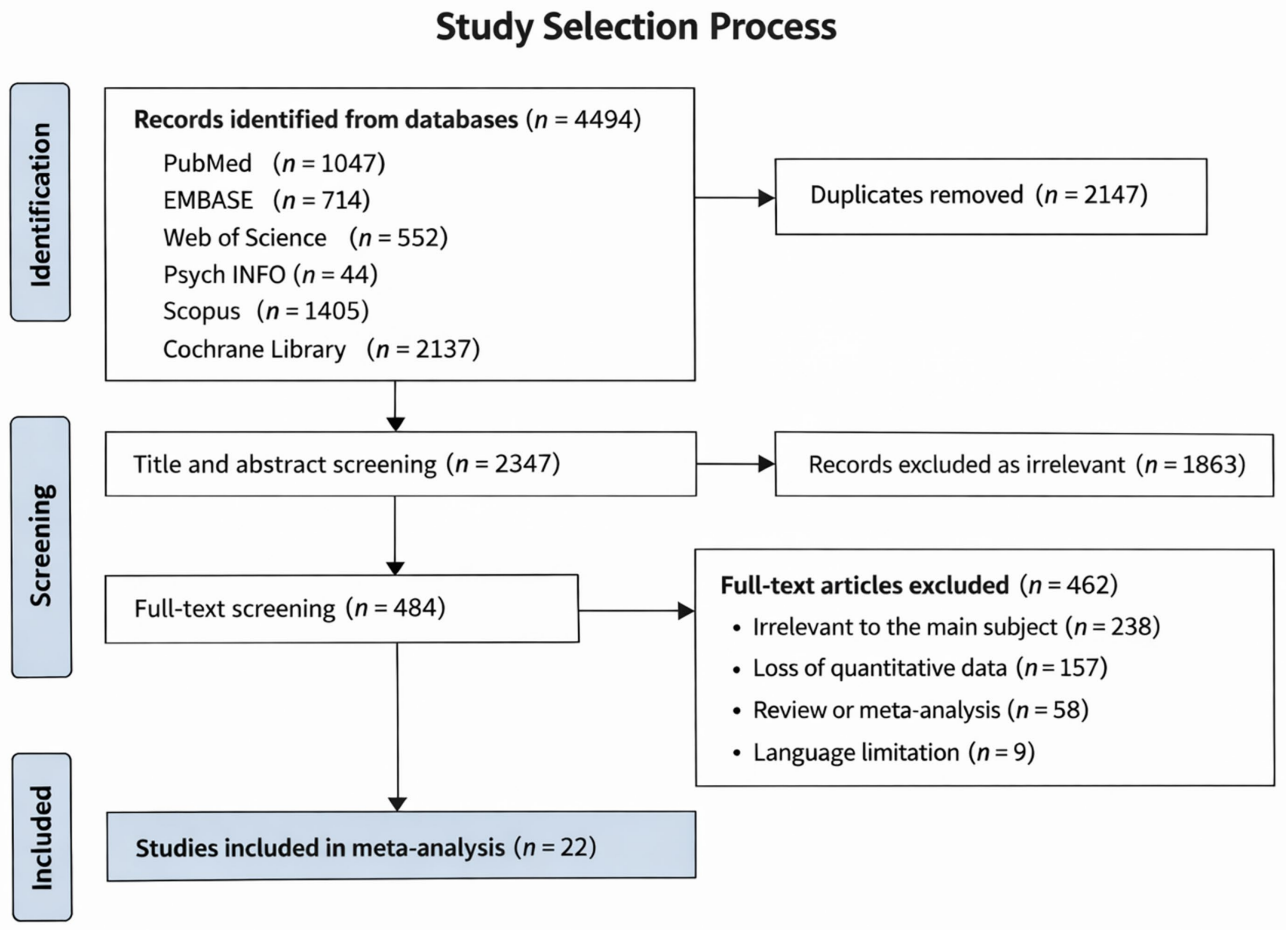
PRISMA flow diagram for the meta-analysis.

No restrictions were applied regarding sample size, geographic origin, or data derivation (Aibibula et al., 2017). Exclusion was applied when studies: (1) utilized non-human subjects; (2) were designed as interventional, laboratory-based, descriptive, review-based, meta-analytic, or case report formats; or (3) contained insufficient quantitative data for effect size estimation. Duplicate data sources were resolved by retaining studies exhibiting maximal follow-up duration or population magnitude. The systematic screening procedure is illustrated in Figure 1.

### 2.3 Data extraction

A standardized data collection form was developed in compliance with the search criteria, with subsequent data extraction and comparison being independently performed by two reviewers (DL and ZC). The extracted items included: title, first author, publication year, geographic region, study design, sample size, offspring’s outcome(s), age at diagnosis, measure of outcomes, type of maternal psychological distress, time of experiencing distress, measure/data source of distress, and measures of association (i.e., adjusted ORs). Duplicate data across publications were excluded, and studies providing the most informative and complete data were preferentially selected. A standardized dataset was systematically established through utilization of the extracted data. Any discrepancies in the process were resolved by discussion with another investigator (YW) or through referring to the original articles (Lin et al., 2019, 2021).

### 2.4 Outcome measure

The outcomes were expressed as ORs, RRs or HRs in eligible studies. The effect sizes were directly used if the original study presented the association between any psychological distress (stress, anxiety, depression, etc.) and the risk of autism or ASD in their offspring (autism or ASD was ascertained using diagnostic criteria such as DSM-V, ICD-10, etc., and the measurements differ across studies). To ensure the maximum comparability of the outcomes across studies, we converted the reported RRs and HRs into ORs.

### 2.5 Critical appraisal for studies included

The methodological quality of all cohort and case–control studies was independently evaluated by two researchers (DL and ZC) through application of the 9-star Newcastle-Ottawa rating system (Wells et al., 2021). A standardized scoring scale of zero to nine was employed, with investigations achieving ≥7 points being categorized as high-quality studies. The methodological quality of included cross-sectional studies was assessed utilizing the 11-item checklist endorsed by the Agency for Healthcare Research and Quality (AHRQ), with investigations achieving ≥8 points being considered as high-quality studies (Rostom et al., 2004; Hu et al., 2015). Critical appraisal disagreements were resolved by consensus.

### 2.6 Data synthesis and statistical analyses

For quantitative synthesis, the effect size along with its 95% confidence intervals (CIs) were extracted from each study. Crude (unadjusted) or adjusted effect sizes were used directly in the pooled meta-analysis calculations. An effect size from a multivariate model was selected when it was generated from both univariate and multivariate models. Forest plots were utilized for summarization of all results, presenting individual effect size estimates with corresponding 95% CIs. Heterogeneity was identified and verified through the *I*^2^ statistic, *Q* test, and *H* statistic (Engels et al., 2000; Higgins and Thompson, 2002; Paz-Bailey et al., 2016). Interpretation of heterogeneity levels was defined as follows: *I*^2^ values of 0–40% were considered potentially unimportant; 30–60% were classified as moderate heterogeneity; 50–90% were categorized as substantial heterogeneity; and 75–100% were designated considerable heterogeneity (Higgins and Green, 2011). Given anticipated substantial heterogeneity, effect sizes were pooled using a DerSimonian and Laird (1986) random-effects model regardless of between-study heterogeneity statistical significance. Funnel plots were generated and meta-bias analyses (Begg’s test, Egger’s test) were performed to examine publication bias (Begg and Mazumdar, 1994; Egger et al., 1997). The stability of conclusions and influence of individual studies were evaluated through application of a one-study removed approach, whereby studies were sequentially omitted to determine whether pooled estimates were substantially influenced by individual studies (Normand, 1999). Between-study heterogeneity was examined through random effects meta-regression analyses, while potential sources of heterogeneity were investigated via predefined subgroup stratification (categorized by study design, autism or ASD measurement methods, maternal psychological distress types, distress assessment, etc.). All analyses were two-tailed, with statistical significance indicated by a *p*-value less than 0.05. Data analyses were performed using Stata version 15.

## 3 Results

### 3.1 Literature search results and study characteristics

A total of 4,494 potentially relevant articles were identified through electronic database searches. Following title and abstract screening, 4,010 records were excluded, resulting in 484 articles being retained for full-text assessment. Ultimately, 22 studies involving 3,164,802 subjects that met all eligibility criteria were included in the final meta-analysis (Zhang et al., 2010; Rai et al., 2012; Hamadé et al., 2013; Hviid et al., 2013; Rai et al., 2013; Visser et al., 2013; Duan et al., 2014; George et al., 2014; Gao et al., 2015; Oerlemans et al., 2016; Hagberg et al., 2018; Chen et al., 2020; Gerges et al., 2020; Krishnan et al., 2021; Avalos et al., 2023; Lin Y. et al., 2023; Nishigori et al., 2023; Mkhitaryan et al., 2024; Seebeck et al., 2024; Tran et al., 2024; Khachadourian et al., 2025; Tusa et al., 2025). The selection process is detailed in Figure 1. Overall, 12½ case–control studies, 8½ cohort studies and one cross-sectional study were included. The included studies were published between 2010 and 2025 and conducted in 11 countries across Europe, Asia, North America, and Oceania, with the largest proportion from Asia (*n* = 11, 50%), followed by Europe (*n* = 7, 32%) and North America (*n* = 3, 14%), and one study from Oceania. Most of them recorded depression (*n* = 11, 50%) and stress (*n* = 10, 45%) as psychological distress during pregnancy. Regarding exposure ascertainment, maternal psychological distress was assessed using non-standardized self-report assessments (*n* = 12, 55%), registry-based or clinically diagnosed distress derived from medical records or registries (*n* = 8, 36%), or standardized psychometric scales (*n* = 2, 9%). Most studies observed the diagnosis of ASD as the health outcome of offspring (*n* = 17, 77%) and the age of autism or ASD diagnosis for offspring ranged from two to 21 years. The measurement of autism or ASD was varied, and the most frequently used ones were ICD-10 (*n* = 6) and DSM-V (*n* = 5). The characteristics of the eligible studies are presented in Table 1.

**Table 1:** Characteristics of studies included in this meta-analysis.

Author (year)	Country	Continent	Study design	Sample size	Children’s outcome(s)	Age at diagnosis (years)	Measurement of outcomes	Type of maternal psychological distress	Time of experiencing distress	Measurement or data source of distress	Distress assessment	Effect size	Quality assessment grade
Avalos et al. (2023)	USA	North America	Cohort	Case: 176 Total: 3,994	Autism-related traits	5.37 ± 2.47	SRS	Depression	From 4 weeks prior to the reported last menstrual period prior to pregnancy through 8 weeks postpartum	Defined by a combination of self-report and diagnoses ascertained from medical records; Diagnosis of depression [International Classification of Diseases (ICD-9)]	Registry-based or clinical diagnosis	aOR	High
Chen et al. (2020)	China	Asia	Cohort	Case: 29,141 Total: 708,515	ASD	4.63 (2.08)	ICD-9	Depression	During pregnancy	ICD-9-CM diagnostic codes of depressive disorder given by board certified psychiatrists	Registry-based or clinical diagnosis	aHR	High
Duan et al. (2014)	China	Asia	Case–control	Case: 286 Control: 286	Autism	Case: 4.26 ± 1.04 Control: 4.14 ± 1.48	DSM-IV, CARS	Anxiety, Stress	During pregnancy	Not mentioned	Non-standardized self-report assessments	aOR	Moderate
Gao et al. (2015)	China	Asia	Case–control	Case: 193 Control: 733	Autism	Case: 9.90 ± 4.39 Control: 9.74 ± 3.73	DSM-IV, CARS	Depression	During pregnancy	Self-reported	Non-standardized self-report assessments	OR	Moderate
George et al. (2014)	India	Asia	Case–control	Case: 143 Control: 200	Autism	Case: 3.50 Control: 3.47	CARS	Stress	Prenatal period	Not mentioned	Non-standardized self-report assessments	aOR	Moderate
Gerges et al. (2020)	Lebanon	Asia	Case–control	Case: 100 Control: 100	ASD	Case: 10.14 ± 5.51 Control: 10.40 ± 5.21	DSM-V	Stress	During pregnancy	Not mentioned	Non-standardized self-report assessments	aOR	Moderate
Hagberg et al. (2018)	UK	Europe	Cohort	Case: 2,154 Total: 194,494	ASD	6.8 ± 3.6	Read diagnostic code	Depression	During pregnancy	Electronic medical database	Registry-based or clinical diagnosis	aRR	High
Hamadé et al. (2013)	Lebanon	Asia	Case–control	Case: 86 Control: 172	ASD	Cases: Boys 12.39 ± 5.92 Girls 10.83 ± 3.23 Controls: Boys 8.09 ± 3.38 Girls 8.45 ± 3.74	DSM-IV-TR	Sadness	During pregnancy	Not mentioned	Non-standardized self-report assessments	aOR	Moderate
Hviid et al. (2013)	Denmark	Europe	Cohort	Case: 3,892 Total: 626,875	ASD	5.6 (4.1–7.5)	ICD-10	Depression	During pregnancy	The National Prescription Registry	Registry-based or clinical diagnosis	aRR	High
Khachadourian et al. (2025)	Denmark	Europe	Cohort	Case: 18,374 Total: 1,131,899	autism	8.3 (5.4–11.8)	ICD-10	Depression	12 months preceding childbirth	The Danish Medical Birth Registry or the Danish Central Population Register	Registry-based or clinical diagnosis	aHR	High
Krishnan et al. (2021)	India	Asia	Case–control	Case: 65 Control: 65	ASD	Case: 4.77 ± 1.57 Control: 4.40 ± 1.52	DSM-V	Stress	Antenatal period	Self-reported	Non-standardized self-report assessments	aOR	High
Lin Y. et al. (2023)	China	Asia	Case–control	Case: 190 Control: 74,356	ASD	Case: 8.20 ± 2.44 Control: 7.59 ± 2.32	DSM-V	Depression	During pregnancy	Self-designed questionnaire	Non-standardized self-report assessments	aOR	High
Mkhitaryan et al. (2024)	USA	North America	Case–control	Case: 168 Control: 329	ASD	3–18	DSM-V	Stress	During pregnancy	Self-reported	Non-standardized self-report assessments	aOR	High
Nishigori et al. (2023)	Japan	Asia	Cohort	Case: 355 Total: 78,745	ASD	3	ICD-10	Depression	The first and the second half of pregnancy	The six-item Kessler Psychological Distress Scale (K6)	Standardized psychometric scales	aOR	High
Oerlemans et al. (2016)	the Netherlands	Europe	Case–control	Case: 288 Control: 480	ASD	11.36 ± 3.64	Autism Spectrum Quotient, Conners Rating Scales, ADI-R	Stress	During pregnancy	Self-designed questionnaire	Non-standardized self-report assessments	OR	High
Rai et al. (2012)	Sweden, England	Europe	Case–control, Cohort	Case: 4,429, Control: 43,277 Case: 73, Total: 11,153	ASD	3–17; 11	ICD-10	Stress	During pregnancy; Early pregnancy - 18 weeks; Mid pregnancy-2 months post delivery	The Swedish Multigenerational Register, the Swedish National Cause of Death Register, the Swedish Cancer Registry, and the National Patient register; Data collected from study mothers on over 40 different common and rare life events at 6 time points covering the prenatal period until 3 years after the child’s birth.	Registry-based or clinical diagnosis	aOR	High
Rai et al. (2013)	Sweden	Europe	Case–control	Case: 4,429 Control: 43,277	ASD	3–17	ICD-10	Depression	During pregnancy	The Stockholm County adult psychiatric outpatient register and the Swedish national patient register	Registry-based or clinical diagnosis	aOR	High
Seebeck et al. (2024)	USA	North America	Cohort	Case: 102 Total: 2,388	ASD	3	SSI-T	Depression; Stress	In the last trimester; During pregnancy	The Edinburgh Depression Scale (EDS); The Psychosocial Hassles Scale (PHS)	Standardized psychometric scales	aOR	High
Tran et al. (2024)	Vietnam	Asia	Cross-sectional	Case: 113 Total: 9,397	ASD	2–6	DSM-V	Stress	During pregnancy	Self-designed questionnaire	Non-standardized self-report assessments	aOR	Moderate
Tusa et al. (2025)	Australia	Oceania	Cohort	Case: 1,443 Total: 223,068	ASD	13–15	ICD-10	Depression	Antenatal period	Diagnosis of maternal perinatal depressive disorders was based on the ICD-10 AM (Australian Modification) utilizing diagnostic codes F32 through F39.	Registry-based or clinical diagnosis	aRR	High
Visser et al. (2013)	the Netherlands	Europe	Case–control	Case: 121 Control: 311	ASD	2.78 ± 0.53	DSM-IV	Stress	During pregnancy	Not mentioned	Non-standardized self-report assessments	aOR	High
Zhang et al. (2010)	China	Asia	Case–control	Case: 95 Control: 95	ASD	3–21	CARS	Unhappy emotional state	During pregnancy	Not mentioned	Non-standardized self-report assessments	aOR	Moderate

### 3.2 Study quality

The quality assessments for studies included in the meta-analysis are presented in Supplementary Tables 1–3. The scores of case–control studies ranged from five to nine and were rated as high quality on average, with a mean of seven according to the NOS; the scores of cohort studies ranged from seven to nine and were rated as high quality on average, with a mean of eight according to the NOS; the score of the cross-sectional study was seven, with a grade of moderate. In all case–control studies, cases and controls were adequately defined, with identical exposure ascertainment methods applied to both groups. However, for over half of these studies, controls were not selected from community or hospital settings, and exposure was not ascertained through secure records or structured interviews blinded to case/control status. All cohort studies included in the meta-analysis were graded as high quality. Non-exposed subjects were drawn from the same population as the exposed cohort, with exposure ascertained through extraction from medical records, electronic medical databases, or registry systems. Confounders were adjusted for exposure ratios, health outcomes were assessed via independent blind evaluation, and subjects were followed sufficiently long for outcomes to manifest. Additionally, study designs ensured autism or ASD in offspring was confirmed absent at baseline (during pregnancy). However, follow-up rates were not reported for some cohorts. In the single cross-sectional study, information sources were clearly defined with listed exposure/exclusion criteria. The pregnancy timeframe was specified, population-based sampling confirmed, objectively measured factors clarified, analytical exclusions explained, and confounding assessment methods described. However, quality assurance procedures, missing data handling protocols, response rate summaries, and follow-up completeness metrics were unreported.

### 3.3 Prenatal maternal psychological distress and the risk of autism or ASD in offspring

**Figure 2:**
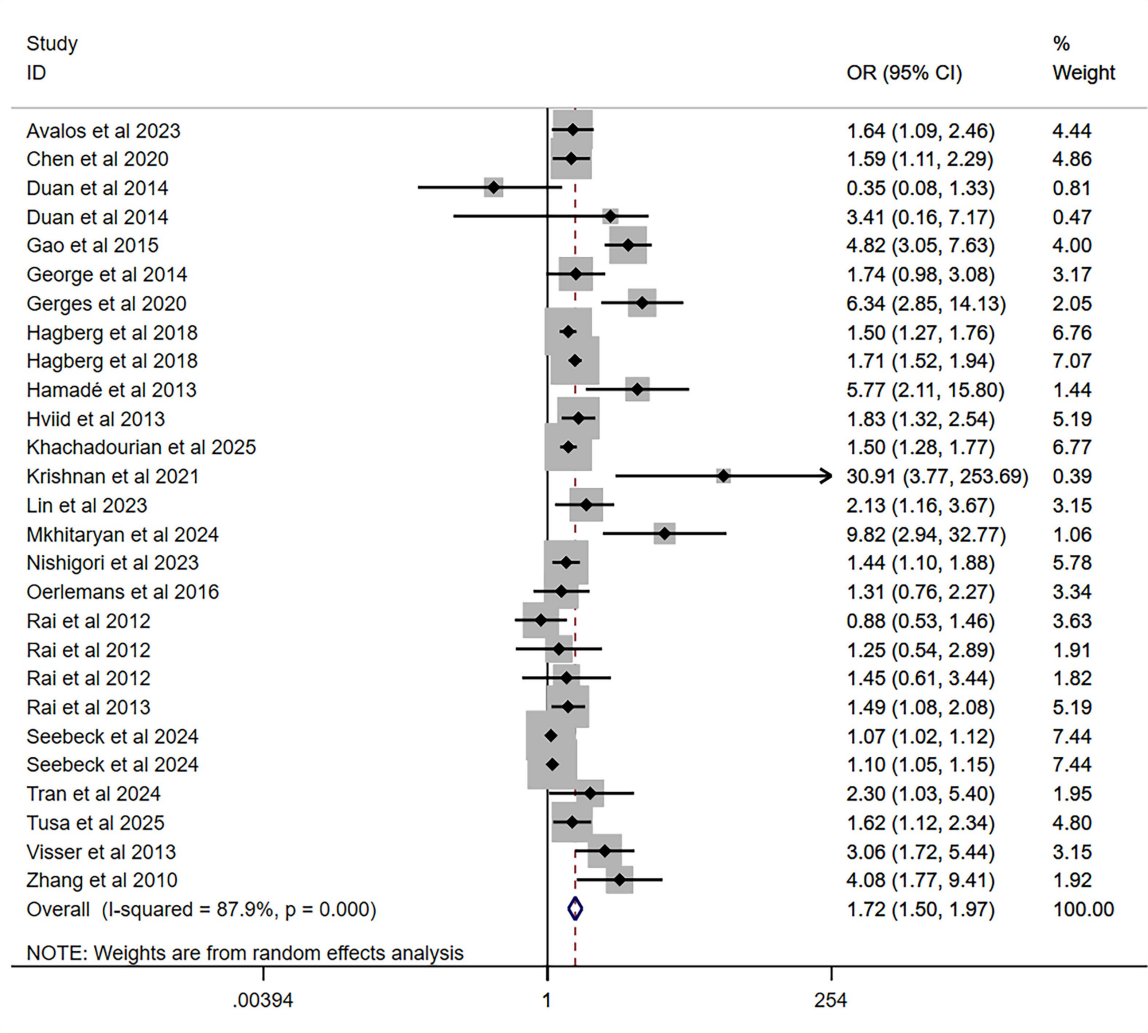
Pooled odds ratio for prenatal maternal psychological distress and the risk of autism or ASD in offspring. One study (Krishnan et al., 2021) reported an unusually large effect estimate with a wide confidence interval (OR = 30.91, 95% CI: 3.77–253.69), which likely reflects the small sample size in that study. Sensitivity analyses confirmed that exclusion of this study did not materially alter the pooled effect estimate.

Twenty-two studies were included in the meta-analysis on the association between prenatal maternal psychological distress and the risk of autism or ASD in offspring. Despite individual study results varying widely from OR = 0.35 (95% CI 0.08–1.33) to OR = 30.91 (95% CI 3.77–253.69), the overall pooled estimate indicated that offspring of mothers with prenatal psychological distress have a 72% increased likelihood of developing ASD or autism (OR = 1.72, 95% CI 1.50–1.97, *p* < 0.01) compared to those of mothers without distress. The pooled association effect sizes (OR values) with their 95% CIs and those from individual studies are presented in Figure 2. The *I*^2^ value was 87.9% (Q = 214.00, *p* < 0.01) and the *H* value was 2.9, which indicated considerable heterogeneity among the studies.

### 3.4 Publication bias

Publication bias was examined by plotting log-transformed association measures (ORs) against their standard errors. Asymmetry was observed through visual inspection of the funnel plot, indicating the presence of publication bias (Supplementary Figure 1). A contour-enhanced funnel plot was employed to aid interpretation (Supplementary Figure 2), and it was demonstrated that six studies had very low statistical significance. Consequently, funnel plot asymmetry was more likely attributed to publication bias. Clear publication bias was further identified by Egger’s linear regression test (*p* < 0.01; Supplementary Figure 3) and Begg’s test (*p* = 0.93; Supplementary Figure 4). Four studies were identified for imputation using the trim-and-fill method (Supplementary Figures 5, 6). Following incorporation of four virtual datasets, meta-analysis was re-conducted, revealing a *Q* value of 237.63 (*p* < 0.01). Application of the random effects model produced a pooled OR of 1.60 (95% CI 1.39–1.82), confirming the robustness of prior findings.

### 3.5 Sensitivity analyses

Leave-one study-out sensitivity analyses were conducted. The pooled ratios for prenatal maternal psychological distress and the risk of autism or ASD in offspring ranged from OR = 1.63 (95% CI 1.43–1.85; when the study by Gao et al. (2015) was excluded) to OR = 1.86 (95% CI 1.56–2.21; when the study by Seebeck et al. (2024) was excluded), suggesting that no study had undue influence on the pooled estimate (Supplementary Figure 7).

### 3.6 Meta-regression and subgroup analyses

Differences among studies were explored and adjusted estimates were generated through meta-regression and subgroup analysis as appropriate. The meta-regression covariates included study design, continent, measurement of autism or ASD, type of psychological distress, and assessment of prenatal maternal psychological distress. The regression models of OR-covariate were established through application of restricted maximum likelihood (REML).

The *p* value of 0.03 was obtained for study design, indicating it was a source of between-study variance. When study design was introduced into the regression model, a tau^2^ value of 0.14 was observed, reflecting improved model goodness of fit. The proportion of between-study variance explained by study design was calculated as 33.25%. Based on the *I*-squared_res output, 84.95% of residual variation was attributed to heterogeneity, with the remaining 15.05% accounted for by within-study sampling variability.

A *p* value of <0.01 was obtained for measurement of autism or ASD, indicating it as a major source of between-study variance. When measurement was incorporated into the regression model, a tau^2^ value <0.01 was recorded, reflecting near-complete improvement in model goodness of fit. The proportion of between-study variance explained by measurement was calculated as 100.00%. Based on *I*-squared_res output, 47.53% of residual variation was attributed to heterogeneity, with the remaining 52.47% accounted for by within-study sampling variability.

A *p* value of 0.004 was obtained for assessment of prenatal maternal psychological distress, indicating it as a substantial source of between-study variance. When distress assessment was incorporated into the regression model, a tau^2^ value of 0.10 was observed, reflecting improved model goodness of fit. The proportion of between-study variance explained by distress assessment was calculated as 48.10%. Based on the *I*-squared_res output, 88.23% of residual variation was attributed to heterogeneity, with the remaining 11.77% accounted for by within-study sampling variability.

When a Knapp-Hartung adjustment was applied, no statistically significant variation in effect estimates was observed across maternal psychological distress subtypes (*p* = 0.78), and this factor did not account for any of the between-study heterogeneity (adjusted *R*^2^ = −12.02%). By comparison, geographic region explained a moderate proportion of heterogeneity (37.85%), although this association did not reach statistical significance (*p* = 0.06). Despite these adjustments, substantial unexplained heterogeneity persisted (*I*-squared_res = 70.7%), indicating that geographic variation alone was insufficient to account for the variability observed across studies.

**Figure 3:**
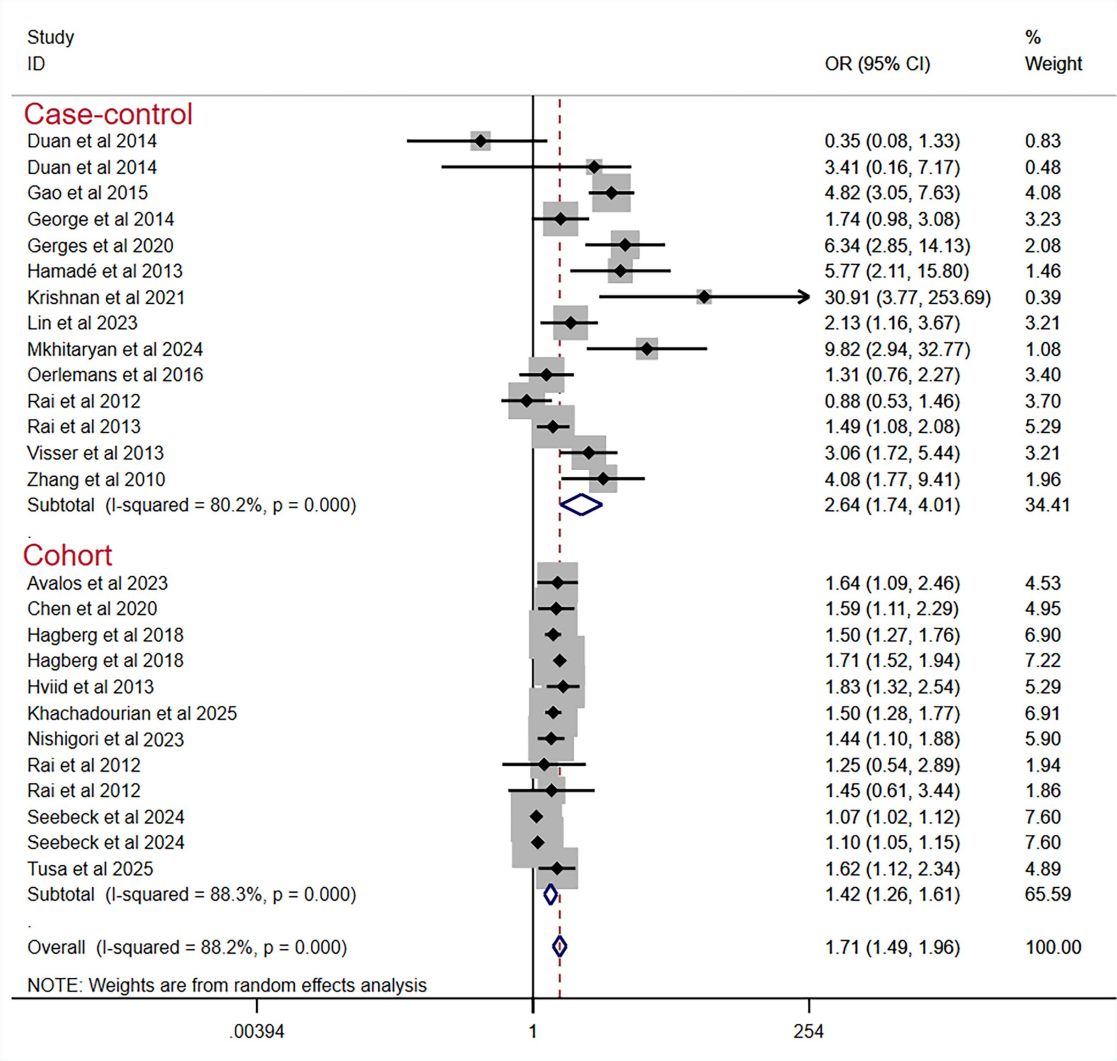
Pooled odds ratio for prenatal maternal psychological distress and the risk of autism or ASD in offspring, categorized by study design.

**Figure 4:**
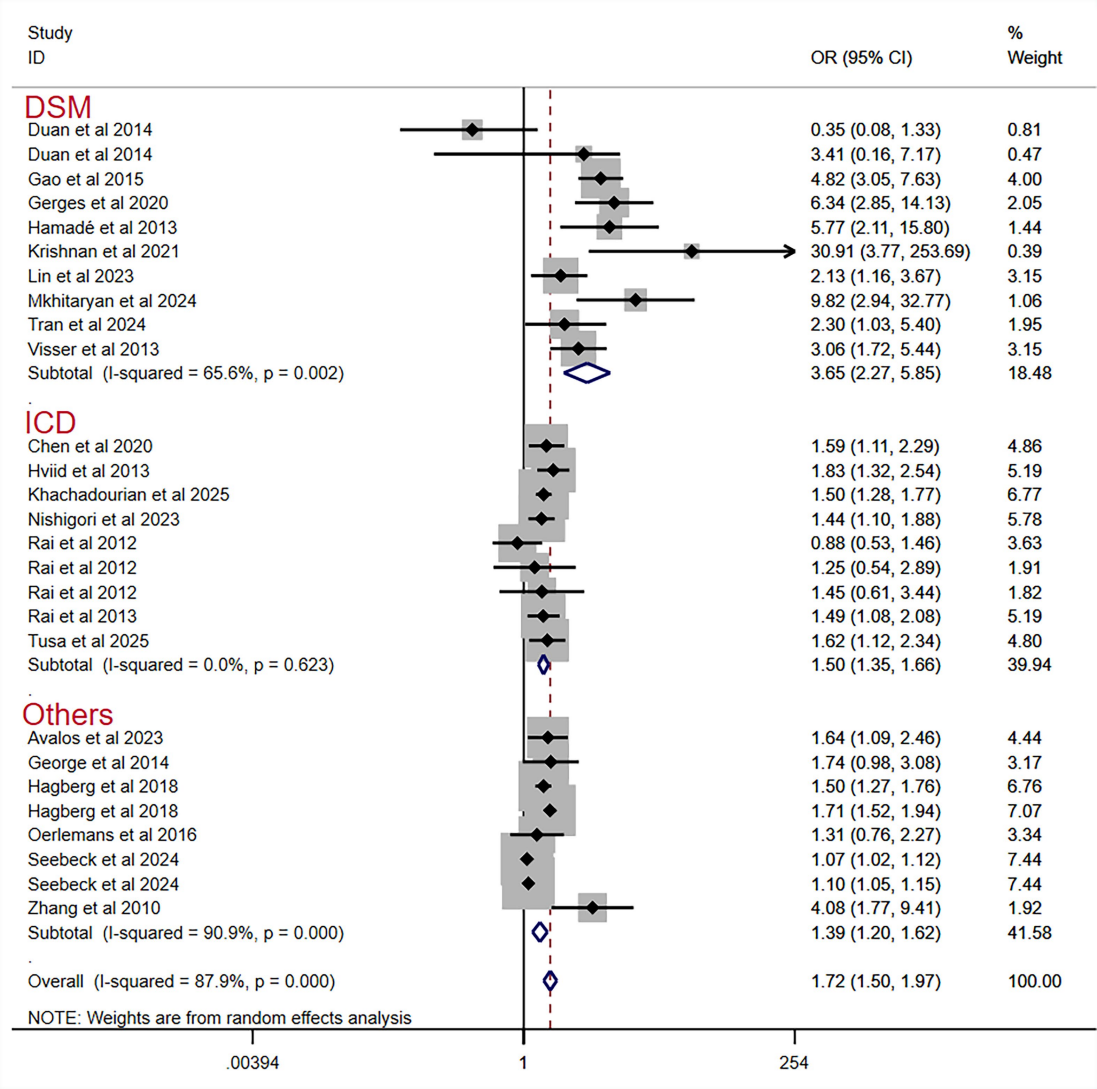
Pooled odds ratio for prenatal maternal psychological distress and the risk of autism or ASD in offspring, categorized by measurement of offspring’s outcome.

**Figure 5:**
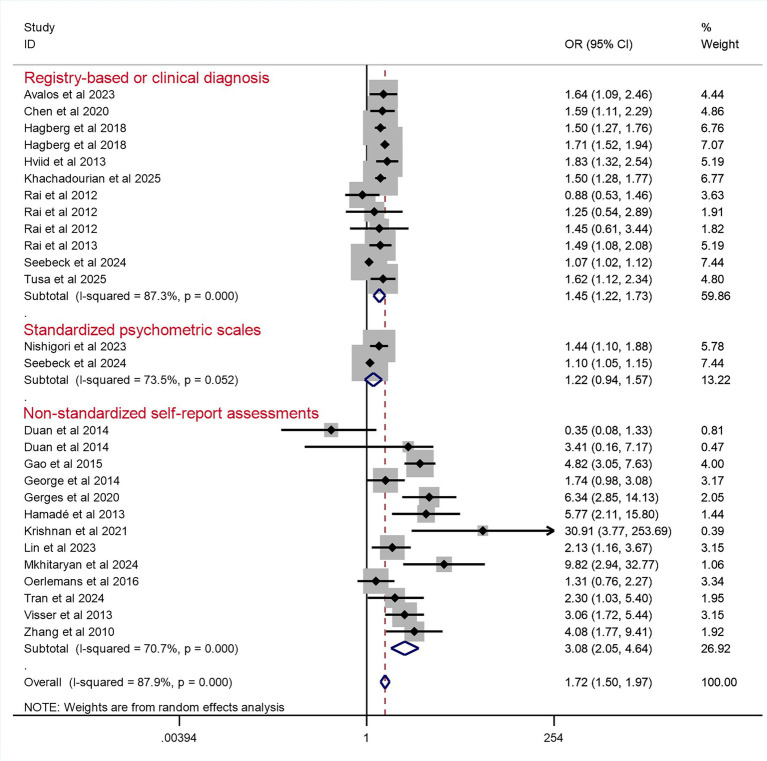
Pooled odds ratio for prenatal maternal psychological distress and the risk of autism or ASD in offspring, categorized by distress assessment.

Hence, we conducted subgroup analyses to recalculate the pooled ORs only according to study design, measurement of autism or ASD, and distress assessment (Table 2 and Figures 3, 4 and 5). Significant associations between prenatal maternal psychological distress and the risk of autism or ASD in offspring were observed in all subgroups. The *I*^2^ test result indicated no heterogeneity in the ICD subgroup [*p* values (*Q* statistic) = 0.62], but there was still considerable heterogeneity among the studies found in other subgroups.

**Table 2:** Odds ratios describing the association between prenatal maternal psychological distress and the risk of autism or ASD in offspring, categorized by subgroups.

Subgroup	Number of effect size	OR	95% CI		Tests for heterogeneity	
					*p* value (*Q* statistic)	*I*^2^ (%)
Study design						
Case-control	14	2.64	1.74	4.01	<0.001	80.2
Cohort	12	1.42	1.26	1.61	<0.001	88.3
Measurement of autism or ASD						
DSM	10	3.65	2.27	5.85	0.002	65.6
ICD	9	1.50	1.35	1.66	0.623	0
Others	8	1.39	1.20	1.62	<0.001	90.9
Distress assessment						
Registry-based or clinical diagnosis	12	1.45	1.22	1.73	<0.001	87.3
Standardized psychometric scales	2	1.22	0.94	1.57	0.052	73.5
Non-standardized self-report assessments	13	3.08	2.05	4.64	<0.001	70.7

## 4 Discussion

This meta-analysis synthesized observational evidence demonstrating a statistically robust 72% increased likelihood of an ASD diagnosis in offspring of mothers experiencing psychological distress during pregnancy. The robustness of this finding was supported by sensitivity analyses showing consistent effect sizes ranging from OR = 1.63–1.86 regardless of individual study exclusion. The pooled OR of 1.72 falls within a range like effect estimates reported for several established neonatal and perinatal ASD risk factors, including low birth weight and neonatal jaundice, as summarized in recent umbrella reviews (e.g., Salehi et al., 2024; Jenabi et al., 2023a). Viewed in this context, prenatal maternal psychological distress does not appear to represent a minor or incidental exposure in epidemiological terms, but rather a risk factor of comparable magnitude within the broader, multifactorial landscape of ASD etiology.

This meta-analysis provides insight into the sources of heterogeneity reported across previous studies. Meta-regression analyses showed that the diagnostic system used to define ASD contributed substantially to the explainable between-study variance, whereas no meaningful variation was attributable to the subtype of maternal psychological distress. Taken together, these results suggest that inconsistencies in the existing literature are more closely related to differences in outcome definition than to true etiological differences across distress categories, highlighting the importance of methodological considerations when interpreting heterogeneous findings. In addition, meta-regression indicated that the method used to assess prenatal maternal psychological distress accounted for a considerable proportion of the between-study variance (48.10%). Studies relying on non-standardized self-report measures tended to report larger effect estimates than those using registry-based or clinically diagnosed exposures, consistent with the possibility that differences in exposure ascertainment contribute to the divergence of reported associations.

Against this methodological background, the observed 72% risk elevation substantially exceeded other modifiable ASD associated factors (pregnancy infections, OR = 1.32; air pollution exposure, OR = 1.20) (Tioleco et al., 2021; Duque-Cartagena et al., 2024). Although cross-exposure comparisons should be interpreted cautiously due to differences in study design and confounding structures, this suggests that maternal psychological distress may represent a potentially impactful and modifiable target for future risk-reduction and supportive research within broader perinatal prevention strategies. This association magnitude matched established obstetric risks (preterm birth, OR = 1.53; gestational diabetes, OR = 1.87) (Crump et al., 2021; Yuan et al., 2024). It nevertheless remained below risk factors with substantially larger effect estimates (extreme prematurity, OR > 5; specific genetic mutations, OR > 10) (Willsey et al., 2022; Yang et al., 2025). These differential risk magnitudes suggested psychological factors likely operate at intermediate tiers within autism’s multifactorial etiology network.

Previous studies and theoretical models have assumed that different types of maternal psychological distress (depression, anxiety, and stress) would constitute an important source of heterogeneity in ASD risk, given their partially distinct neuroendocrine and immunological profiles. However, our meta-regression analyses did not support this assumption. The subtype of maternal psychological distress explained none of the between-study heterogeneity, indicating that the elevated risk of ASD associated with prenatal psychological distress is broadly consistent across diagnostic categories. This finding suggests that, at the level of fetal neurodevelopmental processes, diverse forms of maternal psychopathology may involve overlapping biological pathways, but differential misclassification and confounding across measurement approaches may also obscure subtype-specific differences. In this respect, the likelihood of an autism diagnosis was associated with the presence of maternal distress itself, rather than with specific psychiatric labels assigned to that distress. From an epidemiological perspective, it cautions against over-stratification by psychiatric subtype in future studies, as such distinctions may obscure rather than clarify the underlying signal. Clinically, it may support the use of broad, transdiagnostic psychological screening during pregnancy, rather than relying exclusively on disorder-specific instruments. Such an approach is likely to improve detection, reduce missed cases, and better reflect the dimensional nature of maternal psychological distress as a perinatal risk factor.

Interpretation of these findings should consider potential residual confounding, particularly from genetic and familial factors. Maternal psychological distress is often correlated with parental psychiatric history, which may contribute to both maternal distress during pregnancy and ASD risk in offspring. Evidence from sibling-comparison studies, such as Khachadourian et al. (2025), suggests that shared familial vulnerability may partially account for the observed associations. Moreover, distress experienced during pregnancy frequently persists after delivery and may coincide with variations in the postnatal environment. Because most included studies were observational, separating prenatal from postnatal influences was not feasible. The observed associations therefore likely reflect combined perinatal influences rather than a single causal prenatal effect.

These findings are biologically coherent within the developmental origins of health and disease framework, which posits that prenatal exposures can program long-term neurodevelopment through endocrine and epigenetic mechanisms (Miguel et al., 2019; Chen et al., 2021). Although this meta-analysis could not directly test specific biological pathways, established biological evidence revealed that activation of the maternal stress response system, notably involving the hypothalamic–pituitary–adrenal axis, might allow substances such as glucocorticoids to traverse the placental barrier and disrupt critical phases of fetal brain development (Cottrell and Seckl, 2009; Lautarescu et al., 2020). Animal model studies have shown that prenatal stress has been associated with altered patterns of neuronal connectivity in the prefrontal cortex and changes in hippocampal synaptic plasticity in offspring (Czeh et al., 2018; Namvarpour et al., 2025), which have been linked to neurobiological features associated with ASD. In addition, psychological distress often accompanies behavioral changes such as sleep disorders and irregular diet, which may indirectly affect fetal nutrition supply and circadian rhythm development (Gangitano et al., 2023; Hoyniak et al., 2024). These multiple levels of biological plausibility provide theoretical support for the epidemiological findings of this study, but the exact pathways still need to be elucidated in future research.

From a public health perspective, this discovery has a dual significance. On the one hand, the incidence of psychological distress during pregnancy is significant, with global epidemiological data showing that about 18–25% of pregnant women experience symptoms of psychological distress that meet clinical criteria (Tuksanawes et al., 2020). This means that the potential beneficiaries of intervention measures are large in scale. On the other hand, ASD, as a representative disease of neurodevelopmental disorders, has a high lifelong management cost. The U.S. Centers for Disease Control estimated that each ASD patient needed an average of approximately $2.4 million on medical and social services, while the cost of psychological distress screening and basic interventions (such as cognitive-behavioral therapy) is significantly lower than the long-term care costs for ASD (Buescher et al., 2014). Even if the correlation found in this study was only partially causal, preventive interventions targeting psychological distress during pregnancy may yield meaningful economic benefits at the population level. Analogous cost–benefit considerations have been demonstrated for other perinatal interventions (Detering et al., 2010; Quinn et al., 2024). Although this analysis did not directly evaluate the intervention effect, considering the risk–benefit ratio, these findings support consideration of maternal psychological distress as a priority target for preventive strategies in future interventional and policy studies. However, it should be emphasized that the inherent design limitations of observational studies prevent this study from determining the nature of this association, and residual confounding (such as unmeasured genetic or environmental factors) may still partially explain the observed effects.

From a clinical practice perspective, the findings of this study have enlightening significance for the improvement of prenatal care guidelines and strategies aimed at reducing modifiable risk and supporting neurodevelopment. Currently, in most countries, prenatal examinations mainly focus on monitoring physiological indicators, and systematic screening for mental health has not yet been popularized. For example, although the National Institute for Health and Clinical Excellence (NICE) guidelines recommend screening for depression during pregnancy, the implementation rate is only about 40% (Centre for Mental Health, 2021). Meanwhile, the traditional strategies aimed at reducing modifiable risk and supporting neurodevelopment tend to focus on biological risk factors, such as folate supplementation and infection prevention and control. Our findings underscored the necessity of incorporating maternal mental health into preventive strategies, with particular emphasis on primary healthcare environments. Routine psychological screening for pregnant women in these settings might yield dual advantages, as it could enhance maternal psychological well-being while potentially diminishing neurodevelopmental risks among offspring. Given that registry-based diagnostic systems yielded more stable effect estimates in our meta-regression, embedding standardized mental health screening into routine antenatal records could also strengthen the validity of future population-based ASD surveillance. It was worth noting that the time specificity of this association (limited to the pregnancy stage) supported the critical period hypothesis, which suggested that specific time windows of fetal neural development may be particularly sensitive to changes in maternal psychological states (Adamson et al., 2018; Davis et al., 2020; Lautarescu et al., 2020; Wu et al., 2020).

### 4.1 Limitation

The interpretation of the findings should be considered in the context of the limitations of this study. The representativeness of our analysis is limited for several reasons. First, publication bias was generated by our collection of papers published in English. As with most meta-analyses, the information reported in this source is restricted. Second, there was uniformity of the geographical distribution of the included studies. We have 11 studies from Asia, seven from Europe, but three from North America and one from Oceania, resulting in an unequal weighting of the pooled estimates and making it impossible to generalize our findings to maternal psychological distress in specific contexts or in generally resource-poor settings. Finally, the potential data duplication and geographic concentration in studies may lead to artificially narrow confidence intervals, compromising the validity of statistical inferences. Another limitation is the reporting bias that can be introduced by the different methods used for measuring psychological distress. Twelve out of the 22 studies included in our analysis evaluated psychological distress by non-standardized self-report assessment. Although self-reporting is the standard method of collecting psychological information, it is not as accurate as standardized psychometric assessments, leading to an overestimation of distress level. Furthermore, a considerable proportion of the observed heterogeneity may be explained by differences in diagnostic criteria of autism or ASD, observational periods and study designs (Ioannidis et al., 2008). The absence of consensual choice on measurement of autism or ASD and different observational time frames for the maternal psychological distress was likely to explain some of the variation. In addition, a random effects model was chosen based on the considerable level of heterogeneity in the overall analysis, but substantial heterogeneity remained in the analysis, as is often the case in the meta-analysis of epidemiological studies (Cohn et al., 2011; Wang et al., 2019). In addition, in most included studies, information on maternal psychological distress was only available for the whole period of the pregnancy. Details on the specific trimester of exposure were rarely reported. Consequently, it was not possible to assess whether associations with ASD differed according to the timing of distress during gestation. Finally, although the included studies controlled for many important confounders, such as maternal age, gender of the offspring, race, education level or social support status, it is still possible that there was residual confounding due to the presence of unknown confounders and/or imprecise adjustment strategies (Aibibula et al., 2017). Information on parental psychiatric history and postnatal environmental factors was uneven across the included studies. Variables such as maternal mental health after delivery and early caregiving conditions were not available in a consistent manner, which prevented separate evaluation of their potential influence in this analysis.

## 5 Conclusion

This meta-analysis provides evidence for a modest but statistically significant association between prenatal maternal psychological distress and increased likelihood of an ASD diagnosis in offspring. While the association demonstrates robustness across sensitivity and subgroup analyses, substantial heterogeneity persists due primarily to methodological variations in study design and ASD diagnostic criteria. Our findings support integrating maternal mental health screening into prenatal care frameworks as a component of comprehensive strategies aimed at reducing modifiable risk and supporting neurodevelopment.

## Data Availability

The original contributions presented in the study are included in the article/Supplementary material, further inquiries can be directed to the corresponding author.

## References

[ref1] Adamson B. Letourneau N. Lebel C. (2018). Prenatal maternal anxiety and children's brain structure and function: a systematic review of neuroimaging studies. J. Affect. Disord. 241, 117–126. doi: 10.1016/j.jad.2018.08.029, 30118945

[ref2] Aibibula W. Cox J. Hamelin A. M. McLinden T. Klein M. B. Brassard P. (2017). Association between food insecurity and HIV viral suppression: a systematic review and meta-analysis. AIDS Behav. 21, 754–765. doi: 10.1007/s10461-016-1605-5, 27837425

[ref3] Avalos L. A. Chandran A. Churchill M. L. Gao X. Ames J. L. Nozadi S. S. . (2023). Prenatal depression and risk of child autism-related traits among participants in the environmental influences on child health outcomes program. Autism Res. 16, 1825–1835. doi: 10.1002/aur.2988, 37526980 PMC10857745

[ref4] Begg C. B. Mazumdar M. (1994). Operating characteristics of a rank correlation test for publication bias. Biometrics 50, 1088–1101. doi: 10.2307/2533446, 7786990

[ref5] Biaggi A. Conroy S. Pawlby S. Pariante C. M. (2016). Identifying the women at risk of antenatal anxiety and depression: a systematic review. J. Affect. Disord. 191, 62–77. doi: 10.1016/j.jad.2015.11.014, 26650969 PMC4879174

[ref6] Buescher A. V. Cidav Z. Knapp M. Mandell D. S. (2014). Costs of autism spectrum disorders in the United Kingdom and the United States. JAMA Pediatr. 168, 721–728. doi: 10.1001/jamapediatrics.2014.210, 24911948

[ref7] Centre for Mental Health. (2021). Falling through the gaps: perinatal mental health and the NHS Long Term Plan. Available online at: https://stpsupport.nice.org.uk/perinatal-mental-health/index.html (Accessed January 28, 2026).

[ref8] Champagne F. A. Dosanjh L. H. Firestein M. (2024). “Epigenetic mechanisms linking prenatal maternal stress to developmental outcomes in infants and children” in WAIMH handbook of infant and early childhood mental health: Biopsychosocial factors, volume one. eds. Osofsky J. D. Fitzgerald H. E. Keren M. Puura K. (Cham: Springer International Publishing), 131–145.

[ref9] Chen L. C. Chen M. H. Hsu J. W. Huang K. L. Bai Y. M. Chen T. J. . (2020). Association of parental depression with offspring attention deficit hyperactivity disorder and autism spectrum disorder: a nationwide birth cohort study. J. Affect. Disord. 277, 109–114. doi: 10.1016/j.jad.2020.07.059, 32805586

[ref10] Chen W. Liu N. Shen S. Zhu W. Qiao J. Chang S. . (2021). Fetal growth restriction impairs hippocampal neurogenesis and cognition via Tet1 in offspring. Cell Rep. 37:109912. doi: 10.1016/j.celrep.2021.109912, 34731622

[ref11] Cohn S. E. Jiang H. McCutchan J. A. Koletar S. L. Murphy R. L. Robertson K. R. . (2011). Association of ongoing drug and alcohol use with non-adherence to antiretroviral therapy and higher risk of AIDS and death: results from ACTG 362. AIDS Care 23, 775–785. doi: 10.1080/09540121.2010.525617, 21293986 PMC3095689

[ref12] Cottrell E. C. Seckl J. R. (2009). Prenatal stress, glucocorticoids and the programming of adult disease. Front. Behav. Neurosci. 3:19. doi: 10.3389/neuro.08.019.2009, 19826624 PMC2759372

[ref13] Crump C. Sundquist J. Sundquist K. (2021). Preterm or early term birth and risk of autism. Pediatrics 148:e2020032300. doi: 10.1542/peds.2020-032300, 34380775 PMC9809198

[ref14] Czeh B. Vardya I. Varga Z. Febbraro F. Csabai D. Martis L. S. . (2018). Long-term stress disrupts the structural and functional integrity of GABAergic neuronal networks in the medial prefrontal cortex of rats. Front. Cell. Neurosci. 12:148. doi: 10.3389/fncel.2018.00148 29973870, 29973870 PMC6020798

[ref15] Davis E. P. Hankin B. L. Glynn L. M. Head K. Kim D. J. Sandman C. A. (2020). Prenatal maternal stress, child cortical thickness, and adolescent depressive symptoms. Child Dev. 91, e432–e450. doi: 10.1111/cdev.13252, 31073997

[ref16] DerSimonian R. Laird N. (1986). Meta-analysis in clinical trials. Control. Clin. Trials 7, 177–188. doi: 10.1016/0197-2456(86)90046-2, 3802833

[ref17] Detering K. M. Hancock A. D. Reade M. C. Silvester W. (2010). The impact of advance care planning on end of life care in elderly patients: randomised controlled trial. BMJ 340:c1345. doi: 10.1136/bmj.c1345, 20332506 PMC2844949

[ref18] Dieckmann L. Czamara D. (2024). Epigenetics of prenatal stress in humans: the current research landscape. Clin. Epigenetics 16:20. doi: 10.1186/s13148-024-01635-9, 38308342 PMC10837967

[ref19] Duan G. Yao M. Ma Y. Zhang W. (2014). Perinatal and background risk factors for childhood autism in Central China. Psychiatry Res. 220, 410–417. doi: 10.1016/j.psychres.2014.05.057, 25085792

[ref20] Duque-Cartagena T. Dalla M. D. B. Mundstock E. Neto F. K. Espinoza S. A. R. de Moura S. K. . (2024). Environmental pollutants as risk factors for autism spectrum disorders: a systematic review and meta-analysis of cohort studies. BMC Public Health 24:2388. doi: 10.1186/s12889-024-19742-w, 39223561 PMC11370099

[ref21] Egger M. Davey Smith G. Schneider M. Minder C. (1997). Bias in meta-analysis detected by a simple, graphical test. BMJ 315, 629–634. doi: 10.1136/bmj.315.7109.629, 9310563 PMC2127453

[ref22] Engels E. A. Schmid C. H. Terrin N. Olkin I. Lau J. (2000). Heterogeneity and statistical significance in meta-analysis: an empirical study of 125 meta-analyses. Stat. Med. 19, 1707–1728, 10861773 10.1002/1097-0258(20000715)19:13<1707::aid-sim491>3.0.co;2-p

[ref23] Fombonne E. (2003). Epidemiological surveys of autism and other pervasive developmental disorders: an update. J. Autism Dev. Disord. 33, 365–382. doi: 10.1023/a:1025054610557, 12959416

[ref24] Gangitano E. Baxter M. Voronkov M. Lenzi A. Gnessi L. Ray D. (2023). The interplay between macronutrients and sleep: focus on circadian and homeostatic processes. Front. Nutr. 10:1166699. doi: 10.3389/fnut.2023.1166699, 37680898 PMC10482045

[ref25] Gao L. Xi Q. Q. Wu J. Han Y. Dai W. Su Y. Y. . (2015). Association between prenatal environmental factors and child autism: a case-control study in Tianjin, China. Biomed. Environ. Sci. 28, 642–650. doi: 10.3967/bes2015.090, 26464251

[ref26] Gavin N. I. Gaynes B. N. Lohr K. N. Meltzer-Brody S. Gartlehner G. Swinson T. (2005). Perinatal depression: a systematic review of prevalence and incidence. Obstet. Gynecol. 106, 1071–1083. doi: 10.1097/01.AOG.0000183597.31630.db, 16260528

[ref27] George B. Padmam M. S. R. Nair M. K. C. Leena M. L. Russell P. S. S. (2014). CDC Kerala 13: antenatal, natal and postnatal factors among children (2-6 y) with autism--a case control study. Indian J. Pediatr. 81, 133–137. doi: 10.1007/s12098-014-1594-1 25338492

[ref28] Gerges P. Bitar T. Hawat M. Alameddine A. Soufia M. Andres C. R. . (2020). Risk and protective factors in autism spectrum disorders: a case control study in the Lebanese population. Int. J. Environ. Res. Public Health 17:6265. doi: 10.3390/ijerph17176323, 32878029 PMC7504462

[ref29] Hagberg K. W. Robijn A. L. Jick S. (2018). Maternal depression and antidepressant use during pregnancy and the risk of autism spectrum disorder in offspring. Clin. Epidemiol. 10, 1599–1612. doi: 10.2147/CLEP.S180618, 30464639 PMC6219268

[ref30] Hamadé A. Salameh P. Medlej-Hashim M. Hajj-Moussa E. Saadallah-Zeidan N. Rizk F. (2013). Autism in children and correlates in Lebanon: a pilot case-control study. J. Res. Health Sci. 13, 119–124, 24077467

[ref31] Higgins J. P. T. Green S. (2011). Cochrane handbook for systematic reviews of interventions. Available online at: https://handbook-5-1.cochrane.org/ (Accessed January 28, 2026).

[ref32] Higgins J. P. Thompson S. G. (2002). Quantifying heterogeneity in a meta-analysis. Stat. Med. 21, 1539–1558. doi: 10.1002/sim.1186, 12111919

[ref33] Hoyniak C. P. Whalen D. J. Luby J. L. Barch D. M. Miller J. P. Zhao P. . (2024). Sleep and circadian rhythms during pregnancy, social disadvantage, and alterations in brain development in neonates. Dev. Sci. 27:e13456. doi: 10.1111/desc.13456, 37902111 PMC10997484

[ref34] Hu J. Dong Y. Chen X. Liu Y. Ma D. Liu X. . (2015). Prevalence of suicide attempts among Chinese adolescents: a meta-analysis of cross-sectional studies. Compr. Psychiatry 61, 78–89. doi: 10.1016/j.comppsych.2015.05.001, 26005111

[ref35] Hviid A. Melbye M. Pasternak B. (2013). Use of selective serotonin reuptake inhibitors during pregnancy and risk of autism. N. Engl. J. Med. 369, 2406–2415. doi: 10.1056/NEJMoa1301449, 24350950

[ref36] Ioannidis J. P. Patsopoulos N. A. Rothstein H. R. (2008). Reasons or excuses for avoiding meta-analysis in forest plots. BMJ 336, 1413–1415. doi: 10.1136/bmj.a117, 18566080 PMC2432114

[ref37] Jenabi E. Bashirian S. Salehi A. M. Khazaei S. (2023a). Not breastfeeding and risk of autism spectrum disorders among children: a meta-analysis. Clin. Exp. Pediatr. 66, 28–31. doi: 10.3345/cep.2021.01872, 35879869 PMC9815942

[ref38] Jenabi E. Farashi S. Salehi A. M. Parsapoor H. (2023b). The association between post-term births and autism spectrum disorders: an updated systematic review and meta-analysis. Eur. J. Med. Res. 28:316. doi: 10.1186/s40001-023-01304-2, 37660041 PMC10474756

[ref39] Jenabi E. Salehi A. M. Ayubi E. Seyedi M. Khazaei S. Jourmand H. (2024). Pre and perinatal predictors on autism spectrum disorders: a case-control study in the west of Iran. Matern. Health Neonatol. Perinatol. 10:13. doi: 10.1186/s40748-024-00183-7, 38956743 PMC11220983

[ref40] Kassotaki I. Valsamakis G. Mastorakos G. Grammatopoulos D. K. (2021). Placental CRH as a signal of pregnancy adversity and impact on fetal neurodevelopment. Front. Endocrinol. (Lausanne) 12:714214. doi: 10.3389/fendo.2021.714214, 34408727 PMC8366286

[ref41] Khachadourian V. Arildskov E. S. Grove J. O'Reilly P. F. Buxbaum J. D. Reichenberg A. . (2025). Familial confounding in the associations between maternal health and autism. Nat. Med. 31, 996–1007. doi: 10.1038/s41591-024-03479-5, 39891002 PMC11922763

[ref42] Krishnan V. Krishnakumar P. Gireeshan V. K. George B. Basheer S. (2021). Early social experience and digital-media exposure in children with autism spectrum disorder. Indian J. Pediatr. 88, 793–799. doi: 10.1007/s12098-021-03666-z, 33471317

[ref43] Lautarescu A. Craig M. C. Glover V. (2020). Prenatal stress: effects on fetal and child brain development. Int. Rev. Neurobiol. 150, 17–40. doi: 10.1016/bs.irn.2019.11.002 32204831

[ref44] Lin Y. Wang G. Yang Y. Jin X. Huang H. Zhang Y. . (2023). Risk factors for ASD: risk factors for autism spectrum disorder in Shanghai, China: a population-based case-control study. J. Autism Dev. Disord. 53, 2954–2963. doi: 10.1007/s10803-022-05603-1, 35596026

[ref45] Lin D. Zhang C. Y. He Z. K. Zhao X. D. (2019). How does hard-to-reach status affect antiretroviral therapy adherence in the HIV-infected population? Results from a meta-analysis of observational studies. BMC Public Health 19:789. doi: 10.1186/s12889-019-7135-0, 31221113 PMC6587270

[ref46] Lin D. Zhang C. Shi H. (2021). Effects of clinical pathways on cesarean sections in China: length of stay and direct hospitalization cost based on meta-analysis of randomized controlled trials and controlled clinical trials. Int. J. Environ. Res. Public Health 18:5918. doi: 10.3390/ijerph18115918, 34072956 PMC8198843

[ref47] Lin D. Zhang C. Shi H. (2023). Adverse impact of intimate partner violence against HIV-positive women during pregnancy and post-partum: results from a meta-analysis of observational studies. Trauma Violence Abuse 24, 1624–1639. doi: 10.1177/15248380211073845, 35258353

[ref48] Maenner M. J. Shaw K. A. Baio J. EdS A. Washington A. Patrick M. . (2020). Prevalence of autism spectrum disorder among children aged 8 years - autism and developmental disabilities monitoring network, 11 sites, United States, 2016. MMWR Surveill. Summ. 69, 1–12. doi: 10.15585/mmwr.ss6904a1 PMC7119644 32214087

[ref49] McGowan P. O. Matthews S. G. (2018). Prenatal stress, glucocorticoids, and developmental programming of the stress response. Endocrinology 159, 69–82. doi: 10.1210/en.2017-00896, 29136116

[ref50] Miguel P. M. Pereira L. O. Silveira P. P. Meaney M. J. (2019). Early environmental influences on the development of children's brain structure and function. Dev. Med. Child Neurol. 61, 1127–1133. doi: 10.1111/dmcn.14182, 30740660

[ref51] Mkhitaryan M. Avetisyan T. Mkhoyan A. Avetisyan L. Yenkoyan K. (2024). A case-control study on pre-, peri-, and neonatal risk factors associated with autism spectrum disorder among Armenian children. Sci. Rep. 14:12308. doi: 10.1038/s41598-024-63240-3, 38811666 PMC11137108

[ref52] Moher D. Liberati A. Tetzlaff J. Altman D. G. The PRISMA Group. (2010). Preferred reporting items for systematic reviews and meta-analyses: the PRISMA statement. Int. J. Surg. 8, 336–341. doi: 10.1016/j.ijsu.2010.02.007, 20171303

[ref53] Namvarpour Z. Azhdari S. Lotfi R. Pahang H. Omidi H. Namvarpour M. . (2025). Impact of mild prenatal LPS-induced inflammation and postnatal stress on synaptic plasticity and neuroimmune responses in the prefrontal cortex of adult male mice. Tissue Cell 96:103007. doi: 10.1016/j.tice.2025.103007, 40482402

[ref54] Nishigori T. Hashimoto K. Mori M. Suzuki T. Watanabe M. Imaizumi K. . (2023). Association between maternal prenatal psychological distress and autism spectrum disorder among 3-year-old children: the Japan environment and children's study. J. Dev. Orig. Health Dis. 14, 70–76. doi: 10.1017/S2040174422000411, 35801288

[ref55] Normand S. L. (1999). Meta-analysis: formulating, evaluating, combining, and reporting. Stat. Med. 18, 321–359. doi: 10.1002/(sici)1097-0258(19990215)18:3<>3.0.co;2-p, 10070677

[ref56] Oerlemans A. M. Burmanje M. J. Franke B. Buitelaar J. K. Hartman C. A. Rommelse N. N. J. (2016). Identifying unique versus shared pre- and perinatal risk factors for ASD and ADHD using a simplex-multiplex stratification. J. Abnorm. Child Psychol. 44, 923–935. doi: 10.1007/s10802-015-0081-0, 26466830 PMC4893356

[ref57] Paz-Bailey G. Noble M. Salo K. Tregear S. J. (2016). Prevalence of HIV among U.S. female sex workers: systematic review and meta-analysis. AIDS Behav. 20, 2318–2331. doi: 10.1007/s10461-016-1332-y, 26914165 PMC5114707

[ref58] Quinn M. Halsey J. Sherliker P. Pan H. Chen Z. Bennett D. A. . (2024). Global heterogeneity in folic acid fortification policies and implications for prevention of neural tube defects and stroke: a systematic review. EClinicalMedicine 67:102366. doi: 10.1016/j.eclinm.2023.102366, 38169713 PMC10758734

[ref59] Rai D. Golding J. Magnusson C. Steer C. Lewis G. Dalman C. (2012). Prenatal and early life exposure to stressful life events and risk of autism spectrum disorders: population-based studies in Sweden and England. PLoS One 7:e38893. doi: 10.1371/journal.pone.0038893, 22719977 PMC3374800

[ref60] Rai D. Lee B. K. Dalman C. Golding J. Lewis G. Magnusson C. (2013). Parental depression, maternal antidepressant use during pregnancy, and risk of autism spectrum disorders: population based case-control study. BMJ 346:f2059. doi: 10.1136/bmj.f2059, 23604083 PMC3630989

[ref61] Rostom A. Dubé C. Cranney A. Saloojee N. Sy R. Garritty C. (2004). Celiac disease. Rockville (MD): Agency for Healthcare Research and Quality (US) (Evidence Reports/Technology Assessments, No. 104.) Appendix D. Quality Assessment Forms.

[ref62] Salari N. Rasoulpoor S. Rasoulpoor S. Shohaimi S. Jafarpour S. Abdoli N. . (2022). The global prevalence of autism spectrum disorder: a comprehensive systematic review and meta-analysis. Ital. J. Pediatr. 48:112. doi: 10.1186/s13052-022-01310-w, 35804408 PMC9270782

[ref63] Salehi A. M. Ayubi E. Khazaei S. Jenabi E. Bashirian S. Salimi Z. (2024). Neonatal risk factors associated with autism spectrum disorders: an umbrella review. Clin. Exp. Pediatr. 67, 459–464. doi: 10.3345/cep.2024.00136, 39054642 PMC11374450

[ref64] Seebeck J. Sznajder K. K. Kjerulff K. H. (2024). The association between prenatal psychosocial factors and autism spectrum disorder in offspring at 3 years: a prospective cohort study. Soc. Psychiatry Psychiatr. Epidemiol. 59, 1639–1649. doi: 10.1007/s00127-023-02538-5, 37556019

[ref65] Sevilla L. M. Jimenez-Panizo A. Alegre-Marti A. Estebanez-Perpina E. Caelles C. Perez P. (2021). Glucocorticoid resistance: interference between the glucocorticoid receptor and the MAPK signalling pathways. Int. J. Mol. Sci. 22:10049. doi: 10.3390/ijms221810049, 34576214 PMC8465023

[ref66] Tioleco N. Silberman A. E. Stratigos K. Banerjee-Basu S. Spann M. N. Whitaker A. H. . (2021). Prenatal maternal infection and risk for autism in offspring: a meta-analysis. Autism Res. 14, 1296–1316. doi: 10.1002/aur.2499, 33720503

[ref67] Tran T. T. Nguyen V. T. Nguyen M. P. Huynh Nguyen P. Q. Le T. T. H. Nguyen H. Y. . (2024). Prevalence of autism spectrum disorder diagnosed according to DSM-5 criteria and associated factors in preschoolers in southern Vietnam. Clin. Ter. 175, 405–411. doi: 10.7417/CT.2024.5147, 39584760

[ref68] Tuksanawes P. Kaewkiattikun K. Kerdcharoen N. (2020). Prevalence and associated factors of antenatal depressive symptoms in pregnant women living in an urban area of Thailand. Int. J. Women's Health 12, 849–858. doi: 10.2147/IJWH.S278872, 33116934 PMC7573318

[ref69] Tusa B. S. Alati R. Betts K. Ayano G. Dachew B. (2025). Associations of maternal perinatal depressive disorders with autism spectrum disorder in offspring: findings from a data-linkage cohort study. Aust. N. Z. J. Psychiatry 59, 282–292. doi: 10.1177/00048674251315641, 39895367 PMC11837418

[ref70] Visser J. C. Rommelse N. Vink L. Schrieken M. Oosterling I. J. van der Gaag R. J. . (2013). Narrowly versus broadly defined autism spectrum disorders: differences in pre- and perinatal risk factors. J. Autism Dev. Disord. 43, 1505–1516. doi: 10.1007/s10803-012-1678-6, 23076505

[ref71] Wang Y. Y. Jin Y. Chen C. Zheng W. Wang S. B. Ungvari G. S. . (2019). Meta-analysis of adherence to highly active antiretroviral therapy in patients with HIV infection in China. AIDS Care 31, 913–922. doi: 10.1080/09540121.2018.1554238, 30554523

[ref72] Webster J. C. Oakley R. H. Jewell C. M. Cidlowski J. A. (2001). Proinflammatory cytokines regulate human glucocorticoid receptor gene expression and lead to the accumulation of the dominant negative beta isoform: a mechanism for the generation of glucocorticoid resistance. Proc. Natl. Acad. Sci. USA 98, 6865–6870. doi: 10.1073/pnas.121455098, 11381138 PMC34444

[ref73] Wells G. A. Shea B. O'Connell D. Peterson J. Welch V. Losos M. . (2021). The Newcastle-Ottawa Scale (NOS) for assessing the quality of nonrandomised studies in meta-analyses. Available online at: https://www.ohri.ca/programs/clinical_epidemiology/oxford.asp (Accessed January 28, 2026).

[ref74] Willsey H. R. Willsey A. J. Wang B. State M. W. (2022). Genomics, convergent neuroscience and progress in understanding autism spectrum disorder. Nat. Rev. Neurosci. 23, 323–341. doi: 10.1038/s41583-022-00576-7, 35440779 PMC10693992

[ref75] Woody C. A. Ferrari A. J. Siskind D. J. Whiteford H. A. Harris M. G. (2017). A systematic review and meta-regression of the prevalence and incidence of perinatal depression. J. Affect. Disord. 219, 86–92. doi: 10.1016/j.jad.2017.05.003, 28531848

[ref76] World Health Organization (2022). WHO recommendations on maternal and newborn care for a positive postnatal experience. Geneva: World Health Organization. 35467813

[ref77] Wu Y. Lu Y. C. Jacobs M. Pradhan S. Kapse K. Zhao L. . (2020). Association of prenatal maternal psychological distress with fetal brain growth, metabolism, and cortical maturation. JAMA Netw. Open 3:e1919940. doi: 10.1001/jamanetworkopen.2019.19940, 31995213 PMC6991285

[ref78] Xavier A. M. Anunciato A. K. Rosenstock T. R. Glezer I. (2016). Gene expression control by glucocorticoid receptors during innate immune responses. Front. Endocrinol. (Lausanne) 7:31. doi: 10.3389/fendo.2016.00031, 27148162 PMC4835445

[ref79] Yang B. Zaks N. Kajantie E. Persson M. S. M. Reichenberg A. Gissler M. . (2025). Risk factors for autism spectrum disorder in individuals born preterm: a systematic review and meta-analysis of population-based studies. Biol. Psychiatry Glob. Open Sci. 5:100535. doi: 10.1016/j.bpsgos.2025.100535, 40678694 PMC12268552

[ref80] Yuan J. J. Zhao Y. N. Lan X. Y. Zhang Y. Zhang R. (2024). Prenatal, perinatal and parental risk factors for autism spectrum disorder in China: a case- control study. BMC Psychiatry 24:219. doi: 10.1186/s12888-024-05643-0, 38509469 PMC10956196

[ref81] Zhang X. Lv C.-C. Tian J. Miao R.-J. Xi W. Hertz-Picciotto I. . (2010). Prenatal and perinatal risk factors for autism in China. J. Autism Dev. Disord. 40, 1311–1321. doi: 10.1007/s10803-010-0992-0, 20358271 PMC2974190

