# Supplementary material for: Prenatal maternal psychological distress and the risk of autism spectrum disorders in offspring: results from a meta-analysis of observational studies
Source: Front Psychol. 2026 Apr 20;17:1682620. doi: 10.3389/fpsyg.2026.1682620 (PMC13136244; doi:10.3389/fpsyg.2026.1682620)
Supplement: Supplementary file 1 [file Supplementary_file_1.docx]

Supplementary Material

# Supplementary Tables and Figures

## Supplementary Figures


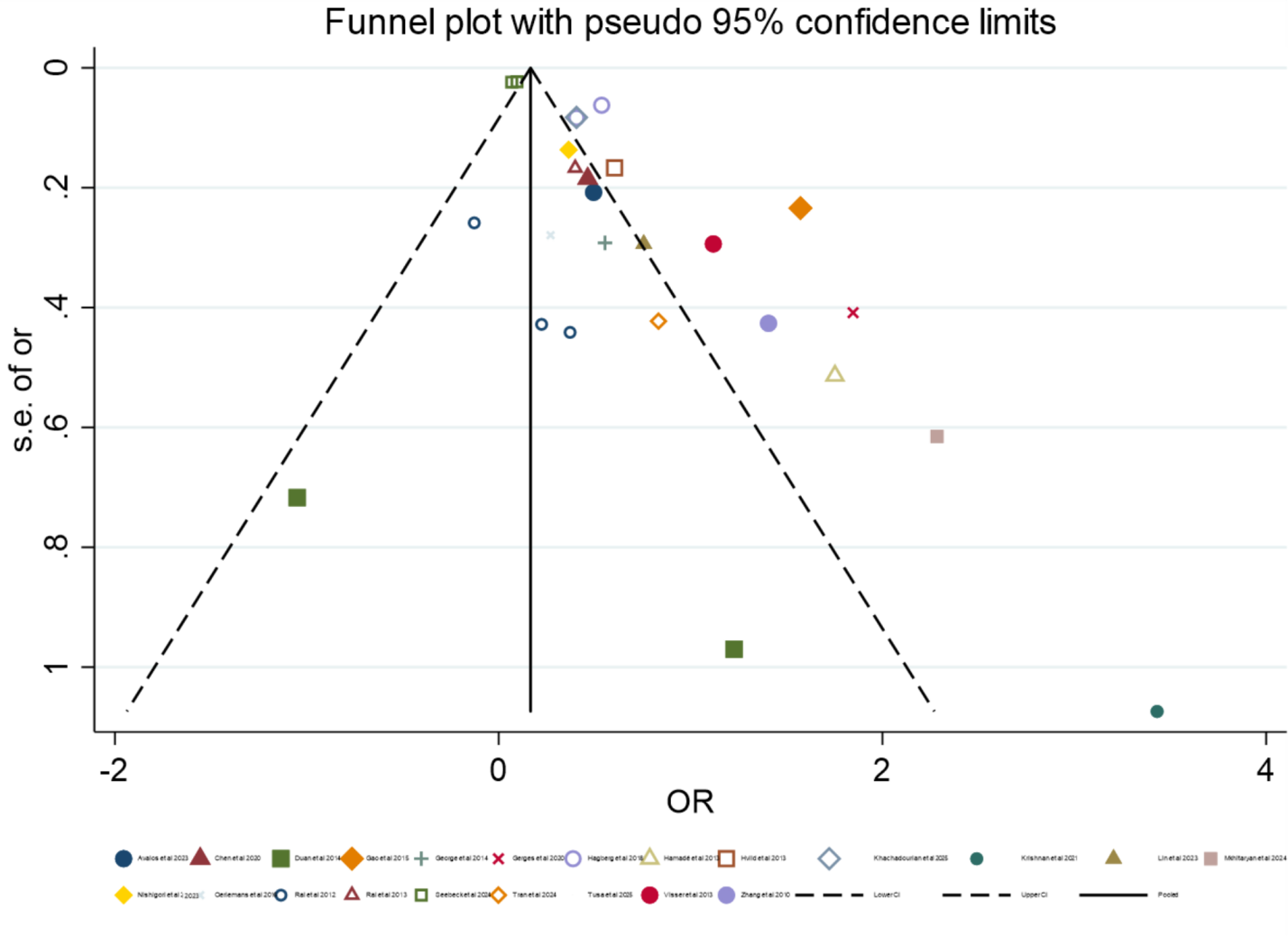


**Supplementary Figure 1.** Funnel plot used to explore the source of publication bias.

**Supplementary Figure 2.** Contour-enhanced funnel plot used to explore the source of publication bias.

**Supplementary Figure 3.** Egger's linear regression test used to explore the source of publication bias.

**Supplementary Figure 4.** Begg's test used to explore the source of publication bias.

**Supplementary Figure 5.** Pooled odds ratio for prenatal maternal psychological distress and the risk of autism or ASD in offspring after four virtual studies were filled.

**Supplementary Figure 6.** Filled funnel plot used to explore the source of publication bias.

**
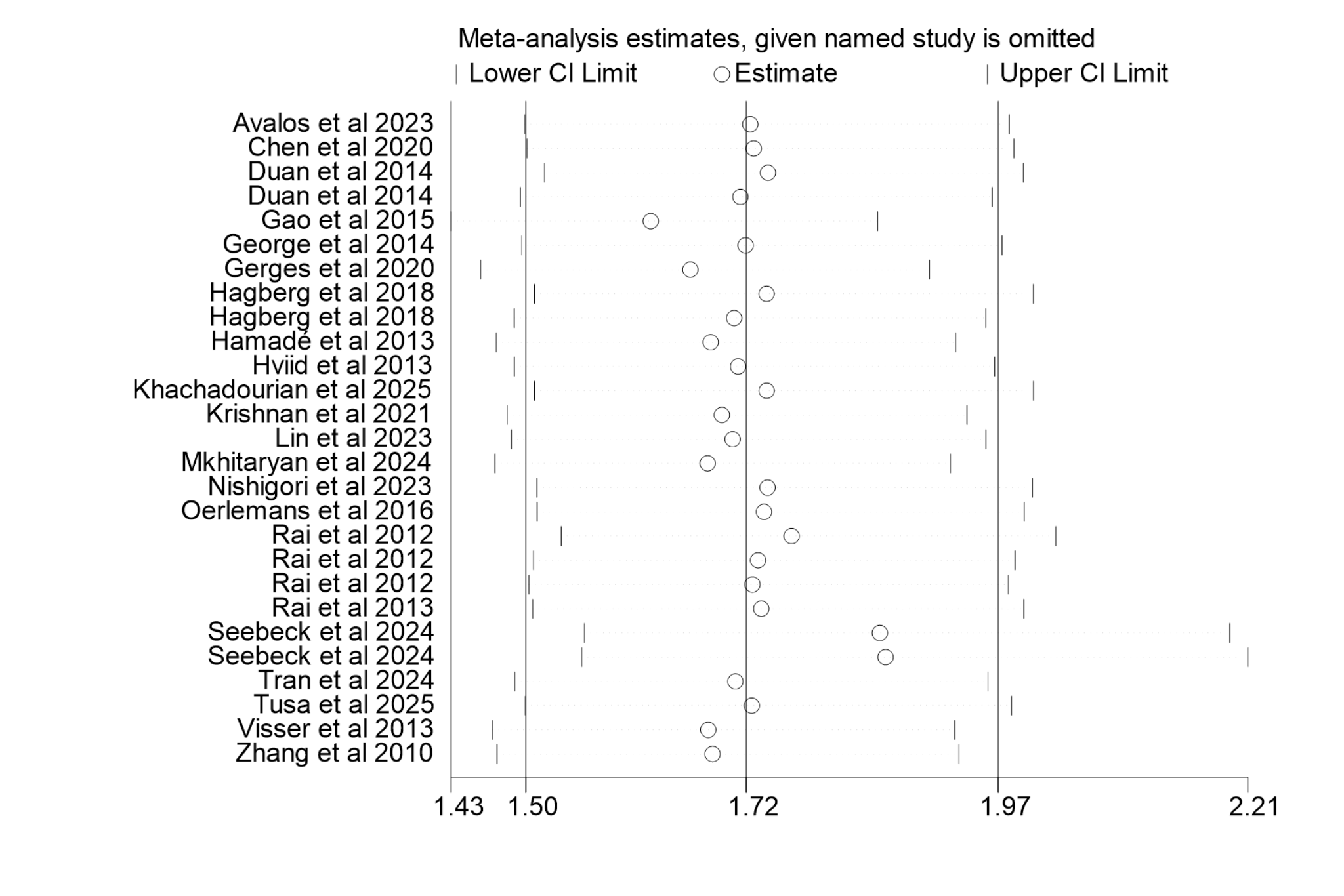
**

**Supplementary Figure 7.** Sensitivity analyses for assessing the impact of individual studies on the pooled estimate.
