# Supplementary material for: Prenatal maternal psychological distress and the risk of autism spectrum disorders in offspring: results from a meta-analysis of observational studies
Source: Front Psychol. 2026 Apr 20;17:1682620. doi: 10.3389/fpsyg.2026.1682620 (PMC13136244; doi:10.3389/fpsyg.2026.1682620)
Supplement: Supplementary file 2 [file Table_1.docx]

Supplementary Material

# Supplementary Tables and Figures

## Supplementary Tables

**Table 1** Results of critical appraisal checklist for cohort studies included in the meta-analysis

| **Study** | **Year** | **Selection** | | | |  | **Comparability** |  | **Outcome** | | | **Quality assessment** | **Grade** |
| --- | --- | --- | --- | --- | --- | --- | --- | --- | --- | --- | --- | --- | --- |
|  |  | Representativeness of the exposed cohort | Selection of the non-exposed cohort | Ascertainment of exposure | Demonstration that outcome of interest was not present at start of study |  | Comparability of cohorts on the basis of the design or analysis |  | Assessment of outcome | Was follow-up long enough for outcomes to occur | Adequacy of follow-up of cohorts |  |  |
| Avalos et al | 2023 | 1 | 1 | 1 | 1 |  | 2 |  | 1 | 1 | 0 | 8 | High |
| Chen et al | 2020 | 1 | 1 | 1 | 1 |  | 2 |  | 1 | 1 | 0 | 8 | High |
| Hagberg et al | 2018 | 1 | 1 | 1 | 1 |  | 2 |  | 1 | 1 | 0 | 8 | High |
| Hviid et al | 2013 | 1 | 1 | 1 | 1 |  | 2 |  | 1 | 1 | 1 | 9 | High |
| Khachadourian et al | 2025 | 1 | 1 | 1 | 1 |  | 2 |  | 1 | 1 | 1 | 9 | High |
| Nishigori et al | 2023 | 1 | 1 | 1 | 1 |  | 2 |  | 1 | 1 | 1 | 9 | High |
| Rai et al | 2012 | 1 | 1 | 1 | 1 |  | 2 |  | 1 | 1 | 1 | 9 | High |
| Seebeck et al | 2024 | 0 | 1 | 1 | 1 |  | 2 |  | 1 | 1 | 1 | 8 | High |
| Tusa et al | 2025 | 0 | 1 | 1 | 1 |  | 2 |  | 1 | 1 | 0 | 7 | High |

**Table 2** Results of critical appraisal checklist for case-control studies included in the meta-analysis

| **Study** | **Year** | **Selection** | | | |  | **Comparability** |  | **Exposure** | | | **Quality assessment** | **Grade** |
| --- | --- | --- | --- | --- | --- | --- | --- | --- | --- | --- | --- | --- | --- |
|  |  | Is the case definition adequate? | Representativeness of the cases | Selection of Controls | Definition of Controls |  | Comparability of cases and controls on the basis of the design or analysis |  | Ascertainment of exposure | Same method of ascertainment for cases and controls | Non-Response rate |  |  |
| Duan et al | 2014 | 1 | 1 | 0 | 1 |  | 2 |  | 0 | 1 | 0 | 6 | Moderate |
| Gao et al | 2015 | 1 | 1 | 0 | 1 |  | 0 |  | 0 | 1 | 1 | 5 | Moderate |
| George et al | 2014 | 1 | 0 | 0 | 1 |  | 2 |  | 0 | 1 | 1 | 6 | Moderate |
| Gerges et al | 2020 | 1 | 0 | 0 | 1 |  | 2 |  | 0 | 1 | 1 | 6 | Moderate |
| Hamadé et al | 2013 | 1 | 0 | 0 | 1 |  | 2 |  | 0 | 1 | 1 | 6 | Moderate |
| Krishnan et al | 2021 | 1 | 1 | 1 | 1 |  | 2 |  | 0 | 1 | 1 | 8 | High |
| Lin et al | 2023 | 1 | 1 | 1 | 1 |  | 2 |  | 0 | 1 | 1 | 8 | High |
| Mkhitaryan et al | 2024 | 1 | 1 | 0 | 1 |  | 2 |  | 0 | 1 | 1 | 7 | High |
| Oerlemans et al | 2016 | 1 | 1 | 1 | 1 |  | 0 |  | 1 | 1 | 1 | 7 | High |
| Rai et al | 2012 | 1 | 1 | 1 | 1 |  | 2 |  | 1 | 1 | 1 | 9 | High |
| Rai et al | 2013 | 1 | 1 | 1 | 1 |  | 2 |  | 1 | 1 | 1 | 9 | High |
| Visser et al | 2013 | 1 | 1 | 1 | 1 |  | 2 |  | 0 | 1 | 1 | 8 | High |
| Zhang et al | 2010 | 1 | 0 | 0 | 1 |  | 2 |  | 0 | 1 | 1 | 6 | Moderate |

**Table 3** Results of critical appraisal checklist for the cross-sectional study included in the meta-analysis

| **Study** | Tran et al |
| --- | --- |
| **Year** | 2024 |
| 1) Define the source of information (survey, record review) | 1 |
| 2) List inclusion and exclusion criteria for exposed and unexposed subjects (cases and controls) or refer to previous publications | 1 |
| 3) Indicate time period used for identifying patients | 1 |
| 4) Indicate whether or not subjects were consecutive if not population-based | 1 |
| 5) Indicate if evaluators of subjective components of study were masked to other aspects of the status of the participants | 1 |
| 6) Describe any assessments undertaken for quality assurance purposes (e.g., test/retest of primary outcome measurements) | 0 |
| 7) Explain any patient exclusions from analysis | 1 |
| 8) Describe how confounding was assessed and/or controlled. | 1 |
| 9) If applicable, explain how missing data were handled in the analysis | 0 |
| 10) Summarize patient response rates and completeness of data collection | 0 |
| 11) Clarify what follow-up, if any, was expected and the percentage of patients for which incomplete data or follow-up was obtained | 0 |
| **Quality assessment** | **7** |
| **Grade** | **Moderate** |

## 
